# The attitudes of mental health professionals towards patients’ desire for children

**DOI:** 10.1186/1472-6939-15-18

**Published:** 2014-03-02

**Authors:** Silvia Krumm, Carmen Checchia, Gisela Badura-Lotter, Reinhold Kilian, Thomas Becker

**Affiliations:** 1 Ulm University, Department of Psychiatry II, Bezirkskrankenhaus Guenzburg, Ludwig-Heilmeyer-Str. 2, 89312 Guenzburg, Germany; 2 Ulm University, Institute of the History, Philosophy and Ethics of Medicine, Frauensteige 6, 89075 Ulm, Germany

**Keywords:** Mental health professionals, Normative orientations, Desire for children, Reproductive autonomy

## Abstract

**Background:**

When a patient with a serious mental illness expresses a desire for children, mental health professionals are faced with an ethical dilemma. To date, little research has been conducted into their strategies for dealing with these issues.

**Methods:**

Seven focus groups with a total of 49 participants from all professional groups active in mental health (nurses, psychologists, social workers and psychiatrists) were conducted in a 330-bed psychiatric hospital. Group discussions were transcribed verbatim and analysed by the documentary method described by Bohnsack.

**Results:**

Mental health professionals did not feel that their patients’ desire for children was as important in daily practice as were parenting issues. When discussing the desire for children on the part of patients, the following themes emerged: “the patient’s own decision”, “neutrality”, “the patient’s well-being”, “issues affecting the children of mentally ill parents” and “appropriate parenthood”. In order to cope with what they perceived as conflicting norms, mental health professionals developed the following (discursive) strategies: "subordination of child welfare", "de-professionalisation", "giving rational advice" and "resignation".

**Conclusions:**

The theme of “reproductive autonomy” dominated mental health professionals’ discourse on the desire for children among psychiatric patients. “Reproductive autonomy” stood in conflict with another important theme (patient’s children). Treating reproductive issues as taboo is the result of the gap between MHPs’ perceptions of (conflicting) norms when dealing with a patient’s desire for children and the limited opportunities to cope with them appropriately.

In order to support both patients with a desire for children and mental health professionals who are charged with providing counselling for such patients, there is a need to encourage ethical reflection and to focus on clinical recommendations in this important area.

## Background

The desire for children is a key issue for many women and men in young adulthood and middle age, irrespective of whether or not they are mentally ill. Awareness of reproductive and parenting issues among mental health service users has increased over the last decade and mental health professionals (MHPs) are urged to consider issues of parenting in their routine practice [1]. However, reproductive issues are likely to confront MHPs with ethical challenges [2]. “Balancing ethical tenets in psychiatric practice is often challenging. These challenges increase in complexity when clinicians must consider simultaneously the needs of a pregnant woman and her fetus, a postpartum woman and her baby, or a woman planning a pregnancy and her not-yet-conceived child” [3]. A number of clinical guidelines and recommendations have been developed for the identification and management of the risk of unwanted pregnancies and the management of pregnancies in patients with severe mental illness [4-6]. According to Coverdale et al. [7] MHPs are in a key position to ameliorate or rectify those factors which impair decision-making capacities in patients whose autonomy is chronically and variably impaired. By balancing the ethical principles of autonomy vs. beneficence, the concept of assisted and surrogate decisions is introduced as a step-wise approach, which includes educating patients on the impact of psychosis on decision making, identification of the patient’s long-standing values and beliefs, and the treatment of psychosis and concomitant physical illness [7]. While these guidelines have been developed to support MPHs’ management of existing pregnancies, there is little literature on providing support and assistance to service users not yet pregnant (or due to be fathers). Literature on the desire for children is mainly focused on psychopharmacological treatment during conception and pregnancy and the peripartal management of mentally ill women during pregnancy or post-partum [8,9]. Frieder [10] provided guidelines on the pre-conception counselling of women with schizophrenia including identification and treatment of risk behaviour, enhancing the patient’s knowledge of the risks to mother and child, improving parenting skills, and mobilizing support systems among women with severe mental illness prior to conception in order to reduce negative outcomes [10]. Despite the existence of these guidelines, there is evidence that professionals do not provide sufficient support and assistance to patients with regard to their desire for children. McLennan and Ganguli [11] showed, based on a survey of clinicians treating patients with severe mental illness at a large community health centre in North Carolina [USA), that clinicians are often unaware of their patients’ birth control status and are unwilling to discuss these issues with patients. Some older anecdotal reports on MHPs suggested that they tend to adopt a negative approach towards their patients’ desire for children [12]. A study of family planning decisions among women with bipolar disorders revealed that most women were insufficiently informed about issues relating to the course of perinatal illness. About half of these women were advised against pregnancy by a (mental) health professional or family member [13].

In their contact with MHPs, mental health service users might feel a need for professional support and assistance in relation to their desire for children. In a German qualitative study on family planning among young women with severe mental illness, female patients reported that their desire for children was hardly ever discussed with MHPs. In the rare event of a discussion of reproductive issues, this was mainly restricted to questions of adequate birth control. Nevertheless, none of the participants reported that they had been advised against motherhood by professionals [14].

Little is known about MPHs’ subjective perspectives on the desire for children among mental health services users. Furthermore, it is not known whether MHPs are aware of the guidelines and recommendations regarding pre-conception counselling and/or the management of pregnancies described above. We assume that the way that MPHs deal with patients’ desire for children is likely to be shaped by individual or collective perspectives on reproductive issues, which, in turn, might be rooted in specific social norms in the form of a set of attitudes, beliefs and expectations that govern behaviour in groups and societies. In order to develop an understanding of MHPs’ normative orientations and strategies when confronted with a patient’s desire for children, the study aims to identify A.) how MPHs talk about their experiences with a desire for children among mental health service users, B.) if and to what extend MHP perceive difficulties and conflicts regarding reproductive issues among mental health service users and C.) MHPs’ discursive strategies to cope with conflicting norms.

## Methods

### Epistemological perspective

The study methodology is based on an exploratory, reconstructive approach. Exploratory approaches are acknowledged as an adequate way to study unexplored fields of research, as they provide an open approach to any issue of interest. Reconstructive methods enable researchers to develop an implicit understanding of the world into explicit rules of understanding. “Understanding”, in a reconstructive approach, is related to two different levels of significance: the first level of significance is apparent in everyday interactions, since social actors are asked to (unconsciously) interpret each other in everyday routine interaction. The second level of significance includes the “habitual practice as well as the ‘objective meaning’ or ‘document meaning’ of particular statements” [15]. Openness is regarded as one of the central components of reconstructive methods as it allows participants to “follow their own form of presentation within their own system of relevance and their own language” [15]. Finally, an open approach reduces the risk of socially desirable comments from participants, because researchers are more reticent regarding their own opinions and perspectives.

### Instruments

Focus groups are regarded as a research method for investigating experiences, attitudes and opinions at the supra-individual level [16]. According to Bohnsack [17], focus groups are appropriate in the study of discursive formations within certain settings. Instead of concentrating on individual perspectives, focus groups are used to explore the products of collective interaction [17]. By discussing specific issues within a “real” group (e.g. MHPs in a clinical setting), group participants refer to each other and produce an interactive process of understanding. Thus, focus groups are a suitable instrument for studying normative orientations among real groups of MHPs.

### Study group

The participants in our study were recruited in a 330-bed psychiatric hospital in a rural area in the south of Germany. Some weeks prior to the beginning of the study, a researcher introduced the study aims and methods to all clinical teams. All professional teams were invited. Out of a total of N = 293 individuals (235 nurses, 27 psychiatrists, 16 social workers, 15 psychologists), a sample of 49 MHPs, including members of all professional groups active in mental health teams (nurses, psychologists, social workers and psychiatrists), were included in the study. We planned to hold eight focus groups (2 sessions per professional group). Since one group did not take place due to organisational problems, we conducted seven focus groups with members of the four professional disciplines (Table 1). While the majority of participants in the study worked in inpatient services, a minority group of participants (n = 7; 14%) were also involved in outpatient services including the outpatient clinic and home treatment teams. 11 out of 49 study participants (22%) were involved in long-term psychotherapy treatment. In total, more than a third of study participants (n = 18, 36%) were concerned with outpatient services and/or long-term psychotherapy (Table 1). Study participants were informed about the aims, content, procedure and voluntary nature of the study. Participants provided oral informed consent to participation in the study. Focus group discussions were analysed anonymously, and confidentiality was ensured. The study was approved by the management board of Bezirkskrankenhaus Guenzburg, data protection commissioner and the hospital’s employee committee. No patients, patient data or patient records were included in the study.

**Table 1:** Focus groups and study participants

	**Round 1**	**Round 2**
Psychologists	July 2011/n = 8	---
	(8 female)	
	*(1 out-patient services*; 1 psychotherapy)	
	d: 55 min	
Social workers	September 2011/n = 5	March 2012/n = 6
	(3 female, 2 male)	(5 female, 1 male)
	d: 75 min	*(1 out-patient services)*
		d: 67 min
Nurses	Oktober 2011/n = 8	February 2012/n = 7
	(5 female, 3 male)	(6 female, 1 male)
	*(2 out-patient services)*	*(7 psychotherapy)*
	d: 115 min	d: 66 min
Psychiatrists	November 2011/n = 6	Dezember 2011/n = 9
	(1 female, 5 male)	(6 female, 3 male)
	*(1 psychotherapy)*	*(3 out-patient services,*
	d: 71 min	*2 psychotherapy)*
		d: 66 min

### Procedure

Data were collected using open and flexible interview guidelines during group sessions. The guidelines were developed in conjunction with a group of researchers (sociologists, psychologists and psychiatrists) and contained items and topics based on the researchers’ pre-existing knowledge (see the ‘Open interview guide’). Each group discussion started with an open introduction on issues of relevance and an invitation to talk about experiences and opinions. A flexible approach to the guidelines allowed the interviewer to adapt to the specific dynamics of the focus groups. Since moderators’ reserve and reticence is an important methodological principle of focus groups, in order to identify the participants’ systems of relevance and in order not to disturb the group’s dynamic, moderators used short and simple questions (or: stimuli) to ensure neutrality or reserve. If no verbal interventions were made by participants, the interviewer encouraged further discussions by referring to the topics in the interview guidelines (see the ‘Open interview guide’). If the participants were sufficiently forthcoming in the discussion and identified their own issues of relevance, the moderator intervened as little as possible.

### **Open interview guide**

1.) Introductive stimuli to generate narratives on MHPs’ experiences

“We are interested in the way mental health professionals are dealing with issues like desire for children or parenthood. We are especially interested in your personal experiences as mental health professionals as well as in your attitudes and opinions in regard to these issues. So, how is it about issues like desire for children or parenthood? Is this a relevant topic? What are your practical experiences?”

2.) “Discourse”

•Experiences, examples

•Relevance of the issues

•Patients’ expectations

•Reproductive issues as clinical tasks

•Clinicians’ responsibility for patients’ reproductive issues

3.) “Problematic areas”

•Children of mentally ill patients

•Birth control

•Childlessness

•Autonomy

•Preventing harm

4.) “Counselling”

Counselling patients regarding reproductive issues

### Analysis

Interviews were transcribed verbatim and given anonymous codes. In the data analysis process we followed the reconstructive approach of the documentary method [15,17]: as a first step (formulating interpretations), two researchers (SK, CC) independently read and structured the text by paramount and sub-ordinated topics. Text passages (subjects, contents, problems) of specific thematic relevance were selected, and (provisional) interpretations were developed. In the second step (reflective interpretation), both researchers compared and contrasted their (provisional) results in a inductive process of theory development. The analysis concentrated on comparisons between different views expressed by group members with regard to selected themes/topics. Alternative meanings were examined by means of comparisons between focus group sessions (using transcripts from different focus groups relating to similar issues) or by relying on researchers’ theories and/or concepts. Finally, central themes and/or categories were summarised and described. The research team (SK, CC, GBL, RK, HF, TB) held regular interpretation sessions in order to discuss the preliminary results in the light of different perspectives. Regular interpretation sessions serve to decrease the risk of researchers providing incorrect subjective interpretations of the data through development of a commonly shared interpretation.

## Results

A) Practical experience among participants with regard to the desire for children

Participants considered discussions between staff and patients regarding the desire for children to be very rare events. Different reasons were thought to account for this. Some focused on the private nature of reproduction, which they thought made this topic less relevant to the psychiatric treatment process; others gave pragmatic reasons, such as limited time resources during a hospital stay, which was considered a barrier to the discussion of wider biographic issues, such as patients’ desire for children.

While most participants shared the view that people with mental illness have the same desire for children as people without mental illness, some participants referred to a specific meaning of a desire for children in a subgroup of people with mental illness. From the point of view of group participants, (future) parenthood among psychiatric patients was sometimes used as a means to achieve normality, to stabilise the living situation, and even as an attempt at self-healing:

“Being a mum means not having to go to work anymore because you are able to stay at home and take care of your children. At long last you have your own family. You have a husband who has to look after you (.) I think that sort of thinking stems from a longing for normality and I think a great number of female patients see it that way.” (Social Workers_1,448).

“And then of course there have been many, many mentally ill parents who have gone down this road in thinking having a child would bring stability into their lives only to discover it brought about severe deterioration for all concerned.” (Psychologists_1,220).

In most cases MHPs talked about female patients’ desire for children, while references to male patients’ reproductive decisions were very rare.

In contrast to the desire for children, parenting issues were seen as more relevant in the mental health care context. Participants referred to more examples and personal experiences with mentally ill mothers and some mentally ill fathers. Participants’ experiences in their clinical caseloads included both positive and negative evaluations of parenting courses. In addition to a wide range of participants’ practical experiences, we found that stories of problematic examples (cases) were contrasted with positive ones, while positive examples were contrasted with problematic ones. Although statements on negative examples (cases) outweighed the positive ones, many participants referred to positive examples of parenthood among psychiatric patients. Interestingly, it appeared that positive examples were often presented as counter to certain expectations of “problematic parenthood”. This means that participants frequently saw positive examples of parenthood among people with mental illness as exceptions, i.e. as deviating from “normality”:

“The whole time I´m trying to think of patients whom I had, where everything went well. In fact, I can remember one female patient we had many years ago, who was psychotic and suffered from depression and was severely ill. Then, we heard she was pregnant and we all said ‘oh my God’. But then, it turned out better than we thought (…) but in most cases with, when I think about it, the female patients we had here who became mothers, it involved a lot of problems.” (Nurses_ 2,420).

B) Normative orientations

### Patient’s own decision

From the perspective of participants, it was naturally always the patient who takes the decision for or against parenthood. These statements were closely linked to the overall principle of patient autonomy as an unquestioned and commonly shared professional value. Indeed, the autonomy principle was neither discussed nor considered:

“But of course, it’s more or less the same with all kinds of decisions: in the end it’s the patient who makes the decision.” (Psychologists _1,336).

The patient’s right to make their own reproductive decisions was frequently linked to historical issues. In fact, throughout all seven groups the discussion arrived at a point where participants referred to the historic context of reproduction among the mentally ill:

“Thank god those times are gone. But I did experience such times, when I first arrived here. There were many patients who underwent forced sterilisation.” (Nurses_1,97).

Patients’ reproductive decisions were related to equality and normality. Participants stressed that a desire for children has to be respected as an equal “right” irrespective whether the patient suffered from mental illness: “Every person has the right to have a child” (Social workers_2,377).

It is noteworthy that only one participant stressed the fact that autonomy could be impaired by mental illness and thus might undermine patients’ ability to take autonomous reproductive decisions. In the following passage, the ability of patients to take autonomous reproductive decisions while experiencing acute psychotic symptoms was called into question:

“Sometimes during an in-patient treatment one might think that it is not a good idea for a female patient to become pregnant (…) how far does our responsibility go here (.) is it possible at all for a patient to make her own decisions in such a case? But apart from that I think it´s (.) I see it as rather uncomplicated. And of course I think as well it has to do with the individual’s point of view, and as far as I´m concerned everyone has the right to happiness and to be able to make decisions regarding whether to have children or not.” (Psychiatrists_1,65).

However, patients’ inability to take autonomous reproductive decisions was presented as an exception rather than the rule.

### Professionals’ neutrality

Participants frequently emphasised a strong obligation to maintain professional neutrality - sometimes in contrast with personal opinion. Some participants talked about situations in which they had to act against their personal convictions in order to meet the expectation of being neutral. In the following passage, a psychologist refers to a discharge situation when she was asked by the female patient whether she was allowed to have another baby. Although the psychologist was strongly concerned about the risk to the child, she felt she was “not allowed” to express her doubts with regard to the appropriateness of (another) pregnancy:

“Well, I did feel rather inhibited (.), because I thought there are certain things I am not allowed to say. I had to be as objective as possible. Although I personally thought it was senseless (to have another child, SK), because there were already two children and the patient was schizophrenic and had already been a threat to these children who had to be taken into care. And, shortly before she was discharged, she asked me if she could have another baby, and I answered her as objectively as I possibly could and that of course it was possible. I told her that if she had to stop her medication then she had to notify us first. But I think I was quite hesitant because, well, I thought of the child’s welfare and asked myself what´s going to happen to these kids? You know? Will they be all right? Who will take care of them?” (Psychologists_1,292)

In some cases participants explicitly dissociated themselves from adopting a negative position. This attempt at distancing themselves is obvious in the following passage from a psychiatrist who subordinates a professional assessment to the concept of a patient’s own decision:

“Of course, none of us are really non-judgmental. That´s a fact. Yes, you can´t - can´t really be. But nevertheless you have to free yourself from that way of thinking. I have to say it’s not about valuations but about patients being able to decide for themselves” (Psychiatrists_1, 586).

Thus, many participants were reluctant to comment on negative evaluations of patients’ reproductive issues. In fact, cases of a negative professional assessment of the desire for children and parenthood were hardly referred to or admitted to among group participants. The only ‘legitimate’ deviation from the rule of “being neutral” was identified in the context of “pro-life argumentation”. This is illustrated by the following passage in which a nurse explicitly refers to a pro-life team decision and thus counteracts the normative orientation towards neutrality:

“Well our team wanted to make pro-life decisions, you know? We thought about how to convince her that such decisions can´t be made overnight and can´t be made from one day to the next in the hope of delaying her decision for as long as an abortion is legally possible” (Nurses_ I, 806).

### Patient’s well-being

MHPs referred to situations in which the (prospective) pregnancy was assessed as likely to have an adverse effect on the course of the patient’s illness, with patient well-being being at stake because of the desire for children. While patients’ well-being is seen as a central part of a professional’s duty to care, interference or the expression of a strong opinion was considered to be contrary to a professional’s neutrality. This is illustrated in the following passage from a psychologist talking about her experience with a female patient who, from the psychologist’s perspective, was likely to be overburdened by her (future) role as a mother:

“Well, it was a planned child and that is what I couldn’t understand: that she made such a decision in her situation. But I would eh, I would, I can´t really express an opinion here. I wouldn´t dare give advice, because I think, well it’s difficult. But I just see that she (the patient) is not well at all and I wonder why she did that to herself” (Psychologists_1,122).

Obviously, the participant was struggling with two contrasting normative orientations: On the one hand, she felt responsible for the patient’s well-being which she considered to be at risk because of the specific demands of motherhood. These “legitimate” doubts were based on the principle of beneficence towards the patient. On the other hand, she was uneasy about expressing these doubts because of the normative expectation of being neutral.

### The issue of the “children of mentally ill parents”

Each discussion included the issue of the problematic situation of the children of mentally ill patients. We identified two types of knowledge from participants’ statements: While some of the participants’ knowledge of the negative effects on children seemed to be based on specific personal experiences with certain patients, many participants referred to a more theoretical and abstract knowledge and were lacking personal experience. Throughout the discussions, there was most agreement on this issue, and individual divergent views were contradicted by the majority of participants.

Overall, the problematic situation of the “children of mentally ill parents” appeared to be a widely shared and accepted “fact”. This fact was considered the norm rather than an exception:

“It is very difficult if a person is at a certain stage in her illness whether schizophrenia, depression or borderline disorder and gives birth to a child. In my opinion bringing a child into the world in a situation like that is to a certain degree irresponsible, because such a child can never receive the maternal care it needs. Well, I know lots of children grow up in inadequate conditions but it´s inevitable that this child will subsequently have mental health issues. I find it very difficult to condone the wish for children among mental patients with long term illnesses.” (Nurses_2,390).

While some participants revealed a rather sceptical view of some psychiatric patients’ parenting abilities, some participants saw mental illness characteristics as diametrically opposed to the requirements of parenthood. Within this line of thought, participants referred to general expectations of “good parenting” (e.g. parental responsibility) which they considered incompatible with suffering from a mental disorder:

“I always think when a child comes into the world it should have maximum opportunities for development, a safe and stable background, really just a (..) stable parental relationship. I think that´s very important for a child. And, yes, it’s a challenging task for mentally ill patients to provide this, it is just much more difficult, you know?” (Social Workers_1,165).

Also, MHPs report on “difficult situations” in which their ideas of appropriate parenthood were undermined:

“I remember a female patient who came to me in the outpatient clinic. And I don’t know (.) she got pregnant by a forensic patient whom she met during her earlier stay in hospital one week before. She was indeed very ill during that time and from my very subjective perspective I think if she hadn´t been ill she wouldn’t have become pregnant. Presumably, it was not her plan to become pregnant at the age of 20 while facing a desperate social and family situation. For me it was a very difficult situation when she came to the outpatient clinic and had no realistic idea about how she was going to care for that child.” (Psychologists _1, 651).

C) Professionals’ strategies for coping with conflicting norms regarding the desire for children

A central aim of the study was to look at professionals’ strategies for handling or coping with conflicting norms. We identified four professional strategies namely:

a) subordination of child well-being issues and/or a decision not to prioritise child well-being;

b) “de-professionalisation” of reproduction issues;

c) information, counselling and “rational advice” and

d) powerlessness and resignation.

a.) **Subordination of child well-being issues and/or a decision not to prioritise child well-being;**

Subordination of child welfare appeared as one strategy to deal with a challenging situation/concurrent relationship between a patient’s reproductive decision and professional knowledge regarding the issue of being a “child of a mentally ill parent”. Against the background of normative orientation towards the patient’s autonomy, some participants tended to subordinate or not prioritise the risk of danger to the child’s welfare. However, rather than denying the negative consequences of parental mental illness in general, these participants stressed the children’s ability to withstand these risks (resilience). The following passage from a psychiatrist illustrates such a line of argument:

Here, the patient’s right to decide upon becoming a mother is ranked above child well-being at the expense of neglecting the latter. The fact that this statement caused a controversial discussion among group participants about the negative effects on children does not counteract but rather corroborates the normative orientation towards the well-being of children.

Some participants stressed the fact that they have a treatment mandate for the adult patient rather than for the patient’s child. From this perspective, patients’ offspring are subordinated to patient well-being. Of course, subordination of child well-being does not imply that child welfare issues are ignored in the treatment process. Rather, the issue of patients’ children is accepted as *one among other* important components within the treatment process. As was said during one focus group session, it is often the patient’s role as a mother (or father) that might be of major relevance in the treatment process. Nevertheless, the main focus remains on the well-being of the (adult) patient, whereas the well-being of the child is of minor relevance:

Finally, another instance of child welfare being subordinated could be found in statements emphasising the positive influences (of parenthood) on the course of maternal illness:

b.) **De-professionalisation” of reproduction**

Many participants emphasised that reproduction is a private issue and thus of minor relevance for psychiatric professionals. Moreover, participants stressed that any professional interference in patients’ reproductive behaviour and/or professional influence on reproductive decisions could be considered a form of ‘invasion of patient privacy’ by staff. In this train of thought, reproduction is an issue which is (and should be) located outside the psychiatric system. Participants constructed a demarcation line between the professional and the private context. This demarcation line was not only a virtual one but could also be identified in physical features, e.g. open doors:

Outside the psychiatric ward reproductive issues were not part of the staff’s responsibility. If at all, they had to rely on “soft” means to convince patients or at least to inform them about appropriate birth control measures. But in the end, professionals lack the opportunities (and do not have the power) to prevent the “normal run of things” (Social Workers_1).

We found that psychiatrists, in particular, discussed in depth the issue of restricted responsibility with regard to parenting issues among patients. One of the main arguments in this discussion was that psychiatrists are obliged not only to meet (medical) professional expectations but that they are overburdened by other expectations:

The participant emphasises his professional responsibility for patients – and not for parents (which is illustrated by participant’s linguistic correction of the term “parent” into “patient”). Patients’ relatives were considered to have an important role in cases of professional scepticism regarding a patient’s desire for children. A psychologist referred to the option of relatives steering and/or taking control of the situation: “Relatives are allowed to say frankly that it is not a very good idea.” (group laughter) (Psychologists_1, 383). Here, group laughter is an indicator of shared knowledge of and agreement with this statement.

De-professionalisation also means that a person’s attitude towards the desire for children and parenting depends on his or her personal point of view: “Well, this is my point of view but this is of course different for everyone.” (Psychiatrists_1, 534).

c.) **Information, counselling and “rational advice**”

Providing appropriate information about risks for the mother and child was considered a legitimate professional strategy. By referring to the concept of (non-directive) counselling, participants explicitly and implicitly focused on patients’ rationality with regard to reproductive decisions. By using this argument participants expressed their hopes that patients would finally take a rational decision *against* motherhood:

Despite the participants’ strong commitment to patients’ reproductive rights and neutrality, there seemed to be room for ambiguous messages. In the following passage, a nurse referred to a situation in which he talked to a patient about her desire for children. Despite an explicit verbal commitment to the patient’s decision, the nurse expressed his own personal opinion regarding not having a child:

Finally, there was a tendency to mask professionals’ reluctance with respect to a patient’s desire for children / reproduction by talking about the “right time” for reproduction in the future. Rather than disapproving of a female patient’s desire for children, many participants talked about their suggestions to ‘postpone’ motherhood or parenthood to a later date:

d.) **Demonstration of powerlessness and resignation**

Many participants stressed the fact that their options for action were rather limited. Thus, taking an explicit powerlessness position could be regarded as another strategy for professionals with regard to their patients’ reproductive issues:

Then, referring to a female patient expressing her desire for children against psychiatric staff’s doubts the participant concluded:

“Everyone is entitled to behave in the way they think is right in the pursuit of personal happiness. And I think, even if a child is not adequately cared for, there are ways and means of dealing with the situation and I would say, I think that children are indeed very robust. They are capable of dealing with such parents or with a mother who is ill.” (Psychiatrists_1, 76).

“Ah, when I get patients like that it´s always a matter of responsibility or getting the patients to take responsibility for their children, which is often a positive approach within the therapy plan. That is the one side of the coin. And the other side of the coin is indeed the worry about the child (…) What happens to the children? The worry about the child is always present. But of course as a rule, the patient has priority. He/she is my patient and even if the child, well the child has second priority here, well of course a child should always come first but it is my patient, and I have to attend to his/her needs. So it’s all about my relationship with the patient and then it suddenly becomes clear and well that of course varies from person to person.” (Psychiatrists_1, 523).

“In my experience even in the most chaotic cases that we have seen in recent years, where mothers decided to have children regardless of constructive advice, something positive turned out in the end. Even in those cases where the children lived in foster care, these (female) patients were more stable and felt more at peace with the world. Even when they had to fight to maintain contact with their children, life was more meaningful for them and more stable.” (Psychiatrists_1,135).

“Well, I think our possibilities are very limited. It’s the way things are done which makes it difficult to interfere. Some months ago we had a female patient who was very ill. It was clear that she would not be in a good state when discharged, simply because her condition would remain the same. And this female patient met a male patient. There were attempts to prevent intercourse on the ward but it was clear that they would try to have intercourse outside the ward. Both patients put pressure on us to speed up their discharge, with the aim of having a child as soon as possible. What did we do? We talked to them about first gaining more stability in their lives, and said that it was too soon and they should get information about family planning. Yes, but (3 sec pause, SK) what else can we do? You have to discharge them at some time.” (Social Workers_1,393).

“Well, I think we have to be careful about extending the boundaries of our field of responsibility. We are not responsible for everything. Well, of course, in the morning, when I get in anyway, our boss asks if the patient’s children are cared for. Clearly, we of course have an additional duty to care for the children. But after all our main duty is to take care of the parents, no, to care for our patients anyway. And I am against that (additional responsibility, SK). Clearly, one can talk about systemic approaches, it’s all about systems, you have to treat everybody, but as far as I´m concerned, I have the patient. And that´s my responsibility. I am not responsible for everything. Therefore, I´m in favour of setting boundaries.” (Psychiatrists_1, 631).

“But I think as far as I´ve experienced it always depends on the counselling process. I think we have to say loud and clear what the risks are. We have to provide information on the medication and so on. But of course I would never advise a person not to become a parent but I would try to counsel them along with doctors, nurses and auxiliaries and all others concerned to talk everything over regarding the risks at stake. ” (Nurses_1,46).

“Due to her social situation, I told her frankly that I thought it would be better for her not to give birth to the child. (…) Well, that was just my opinion. I said: ‘it’s just my opinion, you have to decide for yourself’, because, I would never influence a patient. That’s always bad.” (Nurses_1,834).

“Well, what did we do? We told her that it might not be the right time for it, that it would be better to get more stability, and we talked about contraception, Yes but (…) what else could we do, right?” (Social Workers_1,400).

“Well I think our possibilities for action are rather limited… Actually, I think, it is just natural that one is not allowed to interfere.” (Social Workers_1,380).

“Well, what did we do? We told her that it might not be the right time for it, that it would be better to find some more biographic stability, and we talked about contraception, Yes but (3 sec. pause) what else could we do, right? (laughing). Someday, we have to discharge her (…), simply refer her to other existing services, but well (5 sec. pause), I find, basically, that’s the way it is.” (Social Workers_1,400).

## Discussion

The study aimed at identifying MPHs’ normative orientations regarding reproductive issues among mental health service users and MPHs’ (discursive) strategies for coping with conflicting norms. Based on our study findings, we consider “reproductive autonomy” to be the key normative orientation dominating MPHs’ discourse on the desire for children among psychiatric patients. Although none of the participants explicitly used the term “reproductive autonomy” throughout all seven focus groups, the hypothesis of “reproductive autonomy” as the leading normative orientation could not be disproved by any negative case. “Reproductive autonomy” is usually considered as “the power to decide when, if at all, to have children; also, many - but not all - of the choices relevant to reproduction” [18]: 287]. This concept is mainly debated in the context of the human rights or women’s rights movement and assisted reproductive technologies. Assuming that understandings of “reproductive autonomy” vary depending on context [19], there has as yet been no thorough discussion of its meaning(s) in the context of mental health.

We conceptualise “reproductive autonomy” as the application of the general ethical principle of respect for (patient) autonomy as an important mid-level principle in biomedical ethics. It functions primarily as a principle that protects patients from paternalistic or coercive treatment [20]. “Reproductive autonomy” in the mental health context is rooted in general modernisation processes including increased individual rights to self-determination and autonomy. Patient autonomy as a key value of (post)modern medical cultures is strongly linked to processes of empowerment [21]. Embedded in the Western psychiatric reform movement during recent decades, empowerment in the context of mental health care refers “to the level of choice, influence and control that users of mental health services can exercise over events in their lives” [22]. Our study results indicate that the concept of reproductive autonomy might be part of a particular historical reflection of traditional psychiatric (coercive) approaches to prevent reproduction among psychiatric patients through sterilisation and custodial/paternalistic care for eugenic reasons. By distinguishing professional roles and practice from ‘old’, coercive psychiatry, MHPs position themselves as representatives of a (post)modern mental health care system including respect for patients’ (reproductive) autonomy, equality and normalisation. Participants’ references to neutrality as well as ‘non-directive’ counselling are also in line with the concept of reproductive autonomy.

Against the background of the normative orientation towards (reproductive) autonomy, including neutral or “non directive” professional behaviour, MHPs might consider that they are not “allowed” to interfere with patients’ biographic preferences or to raise concerns about parenthood - even in cases where they have severe doubts as to patients’ ability to provide adequate care for their children. Given that many participants were reluctant to assess patients’ reproductive decisions negatively, we assume that (verbal) restrictive attitudes towards patients’ reproductive freedom are subject to a substantive taboo. This hypothesis is supported by the findings that the only legitimate deviation from the autonomy principle expressed by our participants is a *pro-life* argument.

This taboo seems to stem from two sources: the crimes committed in German Psychiatry during the ‘Nazi era’ and the now widely accepted standards of ethical principles such as respect for autonomy in medicine. The historical experience with eugenic policies and measures not only to increase ‘Arian’ characteristics in the German population and to free hospital beds in war zones but also to reduce costs for the social system by killing ‘useless human beings’ [23] might explain the additional finding that the topic of the ‘costs to society’, i.e. taxpayers, was not mentioned during group discussions. Here, too, we might see the reflections of a specific German taboo currently, that is not to subordinate individual lives and well-being for the ‘sake’ of society. Given the particular historical embeddedness of MPHs’ perspectives, it could be worth studying whether MHPs in Germany are more concerned with the concept of reproductive autonomy than MPHs in other countries.

The well-being of children was found to be another normative orientation of MHPs. Given that almost all participants referred to a (theoretical and practical) knowledge about the problematic situation of patients’ children, the relevance of children’s well-being mirrors current trends within psychiatry to strengthen the status of children of mentally ill parents, as illustrated in a recent guidance “on the protection and promotion of mental health in children of persons with severe mental disorders” by the World Psychiatric Association [24]. The promotion of children’s rights by the UN Convention reinforces MHPs’ responsibility for patients’ children and it seems that MHPs are increasingly aware of patients’ children’s needs [25]. MHPs are asked to consider ‘child well-being’ as an additional, highly valued social norm which increasingly enters the psychiatric system - a system which is primarily aimed at the well-being of the (adult) patient.

Against the background of conflicting normative orientations MHPs are likely to face ethical challenges and dilemmas when confronted with reproductive issues [2,3,7,26]. Based on our study, we found that MHPs developed specific strategies to deal with conflicting normative orientations: first, subordination of child welfare issues and/or non-prioritisation of risks for children appeared to be a “meaningful” coping strategy: concentration on the treatment mandate was found to be an important argument within focus groups for dealing with a patient’s reproductive autonomy. From this perspective, the well-being of children is subordinated to patient well-being. Certainly, subordination of child well-being does not necessarily imply the neglect of issues related to patients’ children in the treatment process. Rather, the issue of patients’ children is accepted as *one among other* important components within treatment processes. In this perspective, subordination of children’s well-being is a secondary effect. This hypothesis is supported by the results of a phenomenological study among mental health nurses showing that a trusting relationship with the client was closely linked to their agreement not to be overly involved with patients’ children [26]. Remaining impartial to the needs of children of mentally ill parents in order not to damage client-therapist relationship could serve as an explanation for the well documented “blind spot” with respect to the children of patients among mental health staff [25,27].

Secondly, participants in our study developed a strategy of “de-professionalisation” and/or “privatisation” of reproductive issues in order to cope with conflicting norms. By confining reproductive issues to patients’ private spheres, MHPs are relieving themselves of professional responsibility for their patients’ reproductive decisions. It is interesting to note that relatives are seen as being not only in a supportive role with regard to parenting issues but also in a “legal” position to interfere with patients’ desire for children. MHPs tend to rely on relatives who are “allowed” to raise severe doubts on the appropriateness of a relative’s desire for children. This finding is in line with results from a study with outpatients which found that one in five mothers perceived that she might lose child custody or visitation rights if she did not adhere to the treatment - 2/3 of them perceived family members as the source of such messages [28]. De-professionalisation also means that MHPs, rather than relying on guidelines or recommendations, refer to their “private” attitudes when dealing with the desire for children. Similarly, in a Finnish cross-sectional study of mental health nurses’ views on clients’ children, it was shown that nurses’ propensity to discuss parenting issues was significantly related to nurses’ personal characteristics such as gender, age, being a parent, and marital status [29].

Thirdly, information and counselling or giving “rational advice” was found to be another professional strategy for coping with conflicting norms. At first sight, counselling is a means of increasing people’s autonomy and corresponds to the autonomy principle. Counselling the patient is consistent with current trends to encourage patients to take responsibility for their own decisions rather than be cared for by professionals. Counselling could also be seen as a (hidden) strategy to curtail professional responsibility and give it back to the patient. Sociologists point to the ambivalent nature of counselling: it provides information and advice, but it does *not* determine what the “right decision” is. Thus, as much as counselling promises help and relief, it might also create uncertainty among subjects [30]. With regard to the focus of our study, counselling appears to be a meaningful strategy for professionals to cope with conflicting normative orientations: relying on patients’ capability to take a “rational” decision *against* parenthood releases MHPs from obligations to interfere with a patient’s desire for children.

However, mental health service users’ autonomy can be (chronically and temporarily) impaired and might have an impact on their desire for children. MHPs might benefit from recommendations and/or clinical guidelines on how to counsel patients in the case of serious concerns about the appropriateness of the desire for children. Coverdale and colleagues introduced recommendations for MHPs to assist patients in decision-making processes [7]. Although not specifically developed for counselling processes regarding the desire for children, central elements of these recommendations might support MPHs in assisting and supporting their patients, including the identification of values and beliefs regarding the desire for children/parenthood. Other approaches emphasise aspects of relational ethics [2,3]: rather than concentrating on a patient’s individual rights, assessment of the specific circumstances of reproductive/parental issues, such as the patient’s social background and relationships, is suggested.

## Conclusions

There seems to be a gap between MHPs’ perceptions of (conflicting) norms when dealing with a patient’s desire for children and the limited opportunities to cope with them appropriately. Many participants view their attitudes towards reproductive issues among their clients as a private matter. “De-professionalisation” and “privatisation” of the desire for children among patients could provide an explanation for MHPs’ reluctance to discuss this issue with their patients. On the one hand, this mirrors MHPs’ respect for the privacy of mental health service users. On the other hand, there might be a risk that patients with a desire for children are counselled or treated arbitrarily by MHPs.

In our study, MHPs did not refer to existing guidelines and recommendations with respect to the identification of patients at risk of “unwanted pregnancies”, management of pregnancies, or preconception counselling [7,10]. Since treating the issue as taboo involves some risks, further development and implementation of specific recommendations for MHPs should be emphasised in order to deal appropriately with a desire for children. Such recommendations should facilitate a more open discourse on this important issue in clinical practice affecting both patients and colleagues. Open discussion could lead to better provision of information to the patients concerned, where not only immediately relevant physiological and medical aspects are addressed, but also uncertainties about the future course of the illness, social and private risks and chances. Assessment of the future skills required to raise children is complex, hypothetical and depends on several circumstances, e.g. the presence of a caring, competent partner, relatives, or the social and financial situation of the parent to be. Such recommendations could help in promoting informed decision-making and reducing the stress and suffering caused by taboos associated with the issue. A first and important step in this process is to encourage MHPs to reflect on their (subjective and collective) views of and attitudes towards reproductive issues among mental health service users. Indeed, at the end of our focus groups, participants expressed their appreciation for this opportunity to encourage them to incorporate these issues into their daily practice in future.

The application of the focus group method has some methodological limitations. Because focus groups aim at discourse including collective orientations and normative meanings, the data gathered are not appropriate for assessing MHPs “real” practices in managing reproductive issues. Also, due to group dynamic processes, the focus group method implies a risk that dominant personalities override dissenting voices. Also, some participants might be reluctant to talk about their concerns regarding reproductive issues among psychiatric patients, e.g. in our study, participants were reluctant to comment on negative assessments of patients’ reproductive issues. Since focus group participants are likely to discuss issues which they regard as ‘accepted statements’ within the group, ‘social desirability’ is part of the research focus. However, we tried to reduce ‘social desirability’ in terms of researchers’ expectations by maximum openness and reservation on the part of researchers from expressing their own opinions and perspectives.

Given the explorative character of our study, with a focus on one hospital with a relatively small number of participants, it does not allow us to make assumptions about MHPs’ attitudes that go beyond our study group. Furthermore, the majority of study participants had been involved in the short-term treatment of hospitalised patients, while only 14% of MHPs had working experience in outpatient/ community services. It is possible that reproductive issues are more relevant for MHPs working in outpatient/ community care. Also, hospital patients might be more severely ill compared to patients in outpatient settings and might thus have provided a distorted picture of MHPs’ attitudes towards reproductive/parenting issues among service users.

## Competing interests

The authors declare that they have no competing interests.

## Authors’ contributions

SK designed and conducted the study, collected and analysed data, drafted the manuscript. CC participated in the design of the study, collected and analysed data, helped to draft the manuscript. GBL participated in the data analysis process and helped to draft the manuscript. RK participated in the data analysis process and helped to draft the manuscript. TB participated in the data analysis process and helped to draft the manuscript. All authors read and approved the final manuscript.

## Pre-publication history

The pre-publication history for this paper can be accessed here:

http://www.biomedcentral.com/1472-6939/15/18/prepub
